# Identification of Differentially Expressed Genes in the Pheromone Glands of Mated and Virgin *Bombyx mori* by Digital Gene Expression Profiling

**DOI:** 10.1371/journal.pone.0111003

**Published:** 2014-10-20

**Authors:** Songdou Zhang, Xiaoming Liu, Bin Zhu, Xinming Yin, Mengfang Du, Qisheng Song, Shiheng An

**Affiliations:** 1 State Key Laboratory of Wheat and Maize Crop Science (College of Plant Protection), Henan Agricultural University, Zhengzhou, Henan, P.R. China; 2 Divisions of Plant Sciences, University of Missouri, Columbia, Missouri, United States of America; Swedish University of Agricultural Sciences, Sweden

## Abstract

**Background:**

Mating decreases female receptivity and terminates sex pheromone production in moths. Although significant progress has been made in elucidating the mating-regulated inactivation of pheromone biosynthesis-activating neuropeptide (PBAN) secretion, little is known about the mating induced gene expression profiles in pheromone glands (PGs). In this study, the associated genes involved in *Bombyx mori* mating were identified through digital gene expression (DGE) profiling and subsequent RNA interference (RNAi) to elucidate the molecular mechanisms underlying the mating-regulated gene expression in PGs.

**Results:**

Eight DGE libraries were constructed from the PGs of mated and virgin females: 1 h mating (M1)/virgin (V1) PGs, 3 h mating (M3)/virgin (V3) PGs, 24 h mating (M24)/virgin (V24) PGs and 48 h mating (M48)/virgin (V48) PGs (M48 and V48). These libraries were used to investigate the gene expression profiles affected by mating. DGE profiling revealed a series of genes showing differential expression in each set of mated and virgin female samples, including immune-associated genes, sex pheromone synthesis-associated genes, juvenile hormone (JH) signal-associated genes, etc. Most interestingly, JH signal was found to be activated by mating. Application of the JH mimics, methoprene to the newly-emerged virgin females leaded to the significant reduction of sex pheromone production. RNAi-mediated knockdown of putative JH receptor gene, *Methoprene tolerant 1* (*Met1*), in female pupa resulted in a significant decrease in sex pheromone production in mature females, suggesting the importance of JH in sex pheromone synthesis.

**Conclusion:**

A series of differentially expressed genes in PGs in response to mating was identified. This study improves our understanding of the role of JH signaling on the mating-elicited termination of sex pheromone production.

## Introduction

Mating in most lepidopteran insects must be precisely regulated because it is limited to a specific photoperiod and developmental stage [1]. Females usually produce and release species-dependent sex pheromones to attract conspecific males for mating. Since the discovery of the first sex pheromone bombykol from the silkworm moth *Bombyx mori*, Sex pheromones have been identified from over 1600 female species [2]. Sex pheromones are synthesized and secreted by pheromone glands (PGs), which are located at the ovipositor tip between the ultimate and penultimate segments of females [3], [4]. Sex pheromones are derived from acetyl-CoA through fatty acid synthesis, desaturation, and chain-shortening reactions followed by the reductive modification of carbonyl carbon [5]. Sex pheromone biosynthesis is precisely regulated by a neuropeptide hormone, namely, pheromone biosynthesis-activating neuropeptide (PBAN) secreted from the subesophageal ganglion in the female head following adult emergence. PBAN directly acts on the PG cells to trigger sex pheromone biosynthesis [6].

Mating elicits a series of behavioral and physiological changes in female adults, for example, it promotes ovarian development, induces oviposition, increases food intake, decreases female receptivity and terminates sex pheromone production [7]–[9]. The temporary and/or permanent termination of female receptivity and suppression of sex pheromone production following mating have been extensively studied on different species. In *Lymantria dispar*, the insertion of the male genitalia into the bursa copulatrix only temporarily suppresses sex pheromone secretion, whereas the presence of sperm in the female spermatheca permanently suppresses this process [10]. The ventral nerve cord (VNC) in *Epipyas postvittan*, exerts an independent pheromonostatic effect, and male factors arrive at a high neural centre via the VNC to suppress pheromone secretion [11]. A pheromonostaitc peptide from male accessory glands can strongly suppress the production of sex pheromone in *Helicoverpa zea* [12]. A sex peptide (SP) released from the male accessory glands of *Drosophila melanogaster* strongly induces the suppression of female receptivity, this SP also stimulates juvenile hormone (JH) synthesis and decreases sex pheromone production in *Helicoverpa armigera* females. This finding suggests that the SP-like factor exerts a stronger effect on the suppression of sex pheromone biosynthesis in *H.armigera* [12], [13]. JH might induce PG response to PBAN stimulation in pharate adults of *H. armigera*, but inhibit the transcript level of PBAN receptor in adult females [13], [14], suggesting that the functions of JH in sex pheromone synthesis possibly vary at different development stages. The PGs in mated *B mori*, females maintain their capability to produce bombykol; however, the suppression of PBAN secretion from the suboesophageal ganglion strongly reduces sex pheromone production [8]. The combination of tactile stimulus and sperm triggers the neuronal inactivation of PBAN release and thus hinders bombykol production after mating [15]. The mechanosensory stimulus and transfer of male factors, including the spermatophore containing sperm and seminal fluid in the corpus bursa suppress sex pheromone release and decrease female receptivity [7], [8].

Therefore, mating directly or indirectly inhibited sex pheromone production. Although PGs are targeted by many mating-produced factors, such as JH and SP, little is known about the effect of mating on expression of genes responsible for sex pheromone production in PGs. In the present study, digital gene expression (DGE) profiling was performed to elucidate the molecular mechanisms underlying the mating-regulated gene expression in PGs of mated and virgin female of *B.mori*.

## Results

### DGE library sequencing

Eight DGE libraries were sequenced [1 h mating (M1)/virgin (V1) PGs, 3 h mating (M3)/virgin (V3) PGs, 24 h mating (M24)/virgin (V24) PGs and 48 h mating (M48)/virgin (V48) PGs], which generated more than 3.5 million raw tags for each of the eight libraries (Table 1). After filtering the adaptor sequences, low-quality tags (tags with unknown nucleotide “N”), empty tags (only adaptor sequence) and tags with a one copy number (probably sequencing error), the total numbers of clean tags still arrived over 3.4 million in each library, and the percentage of clean tags among the raw tags in each library reached over 97.8% (Table 1; Fig. S1).

**Table 1:** Tag analysis statistics.

Summary	V1	M1	V3	M3	V24	M24	V48	M48
Raw Tag	3637787	3680974	3615392	3572205	3738156	3537418	3606992	3623791
Distinct raw tag	132413	131270.5	136329.5	137472	123476	141350	133594	139065
Clean tag	3564408	3606840	3538677	3496245	3669009	3459807	3532683	3544671
Distinct Clean tag	64805	62922	65558	67441	60148	69463	65419	65697
All Tag Mapping to Gene	866805	830485	824184	860504	861588	872023	848985	799383
All Tag Mapping to Gene*	24.31%	23.03%	0.232907	0.24612	23.48%	25.20%	24.03%	22.55%
Distinct AllTag Mappingto Gene	16420	15164	16026	17283	15103	17738	16828	15225
Distinct AllTag Mappingto Gene*	25.34%	24.10%	24.45%	25.63%	25.11%	25.54%	25.72%	23.17%
Unambiguous Tag Mappingto Gene	849418	812002	807144	844560	843660	855176	833944	780344
Unambiguous Tag Mappingto Gene*	23.83%	22.51%	22.81%	24.16%	22.99%	24.72%	23.61%	22.01%
Distinct Unambiguous Tag Mappingto Gene	16124	14892	15745.5	16977.5	14831	17417	16538	14953
Distinct Unambiguous Tag Mappingto Gene*	24.88%	23.67%	24.02%	25.17%	24.66%	25.07%	25.28%	22.76%
All Tag-mapped Genes	5490	5292	5479	5678	5211	5770	5586	5373
All Tag-mapped Genes**	37.55%	36.19%	37.47%	38.83%	35.64%	39.46%	38.20%	36.74%
Unambiguous Tag-mapped Genes	5260	5066	5262	5456	4974	5547	5366	5158
Unambiguous Tag-mapped Genes**	35.97%	34.64%	35.98%	37.32%	34.01%	37.93%	36.70%	35.27%
Mapping to Genome	2174138	2246229	2202715	2130624	2267612	2080664	2180584	2224846
Mapping to Genome*	61.00%	62.28%	62.25%	60.94%	61.80%	60.14%	61.73%	62.77%
Distinct Mapping to Genome	34162	33540	34798	35420	31787	36538	34302	35294
Distinct Mapping to Genome*	52.72%	53.30%	53.08%	52.52%	52.85%	52.60%	52.43%	53.72%
Unknown Tag	523464	530125	511778	505117	539809	507120	503114	520442
Unknown Tag*	14.69%	14.70%	14.46%	14.45%	14.71%	14.66%	14.24%	14.68%
Distinct Unknown Tag	14222.5	14218	14733.5	14738	13258	15187	14289	15178
Distinct Unknown Tag*	21.95%	22.60%	22.47%	21.85%	22.04%	21.86%	21.84%	23.10%

Notes: *presents % of clean tag, **indicates % of ref genes.

The distribution of clean tag expression was analyzed to evaluate the DGE data. The distribution of total clean tags and distinct clean tags in each library showed similar distribution patterns (Fig. S2). The tags with copy numbers of more than 100 consisted of 78% of the total clean tags, but their distribution did not reach 8.0% of the distinct clean tags. In contrast, the low-expression tags with copy numbers of less than 5 accounted for more than 52% of the distinct tag distribution.

### Mapping sequences to the reference genomic data

A total of 60148 to 69463 distinct clean tags were generated in eight libraries, of which 15103 to 17738 could be mapped to genes (Table 1).

**Figure 1:**
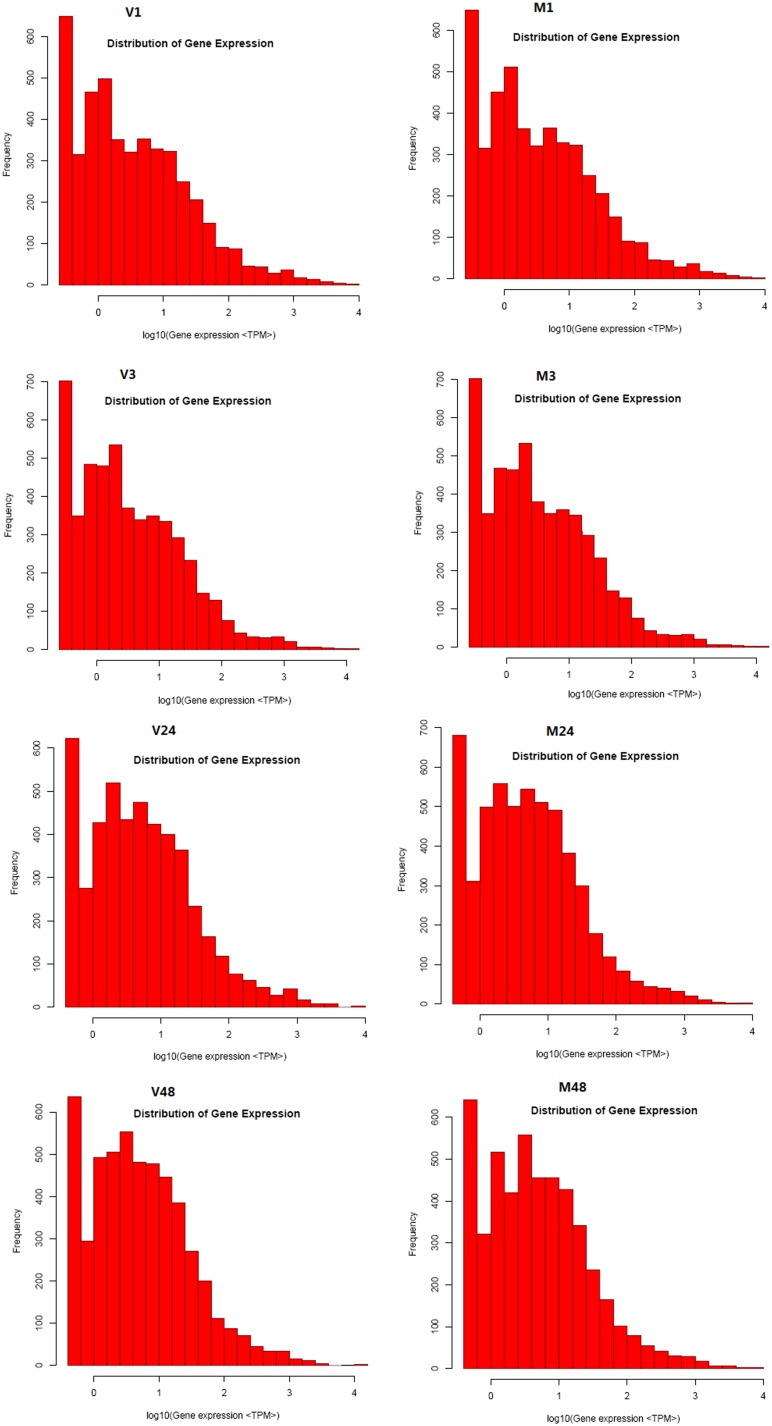
Distribution of gene expression. Gene expression level determined by calculating the number of unambiguous tags and then normalizing to TPM (transcript copies/million tags).

The level of gene expression was analyzed by calculating the number of unambiguous tags for each gene and then normalizing this to the number of transcripts per million clean tags (TPM). As shown in Fig. 1, the majority of transcribed genes were represented in less than 10 copies and only a few genes were highly expressed.

### Analysis of differentially expressed genes in mated and virgin females

**Figure 2:**
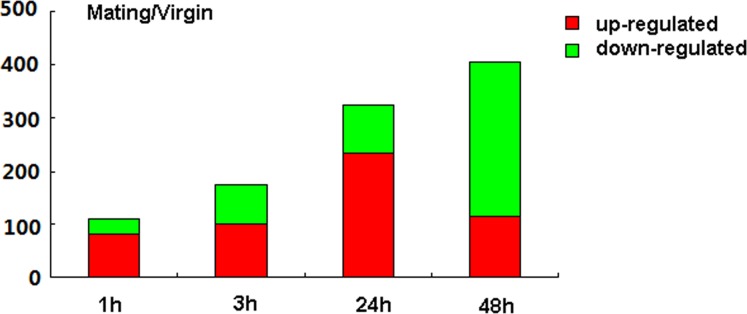
The numbers of differentially expressed genes in each set of mated and virgin female PGs. Up- and down-regulated genes are summarized between M1 and V1, M3 and V3, M24 and V24, and M48 and V48.

The genes that were significantly differentially expressed between the two samples at the same developmental stage were identified following the algorithm developed by Audic *et al.* [18]. A absolute value of log2 ratio ≥1 and *p*≤0.001 were used as thresholds for significant differences in gene expression. The variations in gene expression patterns between M1 and V1, M3 and V3, M24 and V24 and M48 and V48 were analyzed. A total of 111 genes were differentially expressed between M1 and V1 (Fig. 2, Fig. S3 and Table S1), with 81 up-regulated and 30 down-regulated genes. GO classification analysis revealed that these differentially expressed genes were correlated with metabolic process and response to stimulus, signaling etc (Fig. S4).

The comparative analysis of M3 and V3 libraries revealed that 174 genes were differentially expressed with 101 up-regulated genes and 73 down-regulated genes (Fig. 2, Fig. S3 and Table S2). GO classification analysis revealed that these differentially expressed genes were associated with many biological process such as development process, metabolic process, regulation of biological process, signaling, response to stimulus and so on (Fig. S4).

A total of 325 genes showed significant changes between M24 and V24, including 235 up-regulated and 90 down-regulated genes (Fig. 2, Fig. S3 and Table S3). GO classification analysis revealed that these differentially expressed genes were correlated with developmental process, metabolic process, signaling, growth, reproduction and so on (Fig. S4).

A comparison of M48 and V48 revealed 114 up-regulated genes and 292 down-regulated genes (Fig. 2, Fig. S3 and Table S4). Further GO analysis revealed that these genes were associated with developmental process, immune system process, metabolic process signaling and so on (Fig. S4).

A series of immune-associated genes was rapidly up-regulated at 1 h after mating. These genes include those which encoding gloverin family, attacin, lebocin-3, antimicrobial protein 6 Tox, protease inhibitor 6, lysozyme, peptidoglycan recognition protein, beta-1, 3-glucan recognition protein 2, and so on (Table 2). JH signal-associated genes, such as JH bind protein, were also up-regulated at early mating stages (1 h and 3 h). *Krüppel homolog 1*, a JH-mediated transcript factor, was significantly expressed 24 h after mating. This finding suggests that JH is crucial in mating-induced physiological response. In addition, genes involved in the fatty acid/triacylglycerols (TAG) synthesis and metabolism were induced immediately after mating, remained at high level until 24 h after mating, and then deceased 48 h after mating (Table 2). These results suggest that PGs are still capable for sex pheromone synthesis and release even after mating.

**Table 2:** Differential expression genes associated with several pathways.

Gene ID	Gene name	M1/V1	M3/V3	M24/V24	M48/V48
**Immune-associated genes**					
BGIBMGA013803-TA	gloverin 3	**+**			
BGIBMGA002747-TA	attacin	**+**			
BGIBMGA005658-TA	Gloverin 2	**+**			
BGIBMGA000861-TA	Antimicrobial protein 6 Tox	**+**			
BGIBMGA002739-TA	attacin	**+**			
BGIBMGA004869-TA	Protease inhibitor 6	**+**			
BGIBMGA013865-TA	Gloverin 6	**+**			
BGIBMGA010439-TA	lysozyme	**+**			
BGIBMGA008038-TA	peptidoglycan recognition protein	**+**	**+**	**−**	
BGIBMGA009953-TA	serine protease inhibitor 28	**+**	**+**		
BGIBMGA004727-TA	protease inhibitor 3	**+**	**+**		
BGIBMGA004728-TA	serine protease inhibitor 012	**+**	**+**		
BGIBMGA011687-TA	putative leukocyte receptor cluster member	**+**	**+**		
BGIBMGA011609-TA	beta-1,3-glucan recognition protein 2	**+**			
BGIBMGA006775-TA	Lebocin-3	**+**	**−**	**−**	
BGIBMGA009072-TA	fungal protease inhibitor F	**+**			
BGIBMGA008711-TA	protease inhibitor 4		**−**		
BGIBMGA011574-TA	protease inhibitor 1			**+**	
BGIBMGA013958-TA	serine protease inhibitor 14			−	
BGIBMGA010212-TA	serine protease inhibitor 3			−	
BGIBMGA000023-TA	cecropin-B			**−**	**+**
BGIBMGA013746-TA	prophenoloxidase activating enzyme			**−**	
BGIBMGA008247-TA	Lectin5			**−**	**−**
BGIBMGA012264-TA	lysozyme				**−**
**Fatty acid and TCA synthesis and metabolism associated genes**					
BGIBMGA008440-TA	aldehyde oxidase 2	**+**	**+**		
BGIBMGA007880-PA	acyltransferase	**+**			
BGIBMGA006185-TA	fatty acid transport protein	**+**	**+**		
BGIBMGA002160-TA	fatty-acyl CoA reductase 6		**+**		
BGIBMGA014031-TA	alcohol dehydrogenase		**+**		
BGIBMGA007838-TA	acyltransferase		**+**		
BGIBMGA008960-TA	lipase		**+**		
BGIBMGA008049-TA	diacylglycerol o-acyltransferase		**+**	**+**	
BGIBMGA013593-TA	perilipin		**+**		
BGIBMGA005695-TA	triacylglycerol lipase		**−**		
BGIBMGA004655-TA	fatty acid synthase			**+**	
BGIBMGA002940-TA	alcohol dehydrogenase			**+**	
BGIBMGA006603-TA	sarco/endoplasmic reticulum calcium ATPase			**+**	
BGIBMGA008439-TA	aldehyde oxidase 1			**+**	
BGIBMGA007490-TA	glyceraldehyde-3-phosphate dehydrogenase				**−**
BGIBMGA000715-TA	acyl-CoA dehydrogenase				**−**
BGIBMGA007361-TA	3-hydroxyacyl-CoA dehydrogenase				**−**
BGIBMGA012635-TA	acyl-CoA-binding domain-containing protein 6-like				**−**
BGIBMGA011148-TA	fatty-acyl CoA reductase 3				**−**
BGIBMGA011563-TA	acyl-CoA delta-11 desaturase/conjugase				**−**
**JH signal associated genes**					
BGIBMGA003404-TA	JHBP like protein	**+**	+		
BGIBMGA001926-TA	farnesyl diphosphate synthase		+		
BGIBMGA003160-TA	Kruppel homolog 1			**+**	
BGIBMGA003345-TA	juvenile hormone binding protein brP-1649			**+**	
BGIBMGA013930-TA	juvenile hormone epoxide hydrolase			**+**	
BGIBMGA006839-TA	nuclear hormone receptor E75 isoform B			**+**	

Notes: + presents up-regulation, − indicates down-regulation.

### Effect of JH on bombykol production

**Figure 3:**
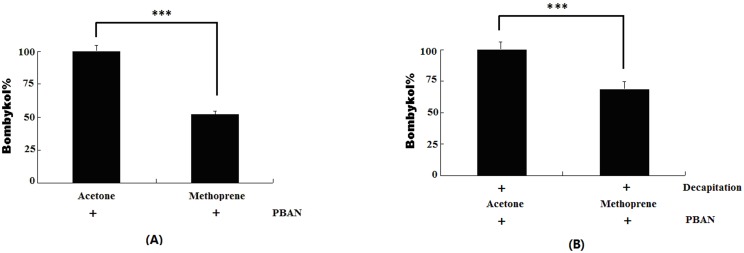
The effect of JH on bombykol production. (**A**) Newly emerged virgin females were treated with JH analogues (Methoprene) for 2 h, followed by injection of 10 pmol PBAN for 90 min. The acetone was used as negative control. The PGs then were removed and extracted in hexane for further GC/MS analysis. (**B**) Newly emerged virgin females were decapitated immediately after emergence. After a 24 h waiting period, the decapitated females were treated with methoprene or acetone (control) and PBAN before PG dissection and extraction for GC/MS analysis as described above. Bars indicate the mean values ± S.D. for independent experimental animals (n> = 12). Statistically significant differences from the PBAN alone are denoted by *** (*p*<0.01) as determined by the Student’s *t*-test.

Gas chromatography/mass spectrometry (GC/MS) analysis revealed that when the newly emerged virgin females were treated with JH analog methoprene, bombykol level was significantly decreased (Fig. 3A), suggesting that JH significantly inhibited bombykol production in newly emerged females. Similar results were observed in decapitated females, in which the newly emerged females were decapitated, allowed to stay for 24 h to exclude the endogenous PBAN, and then treated with JH and PBAN (Fig. 3B). These results clearly show JH treatment led to significant reduction of bombykol production in newly emerged females.

### Expression analysis of methoprene-tolerant (*Met*) genes

**Figure 4:**
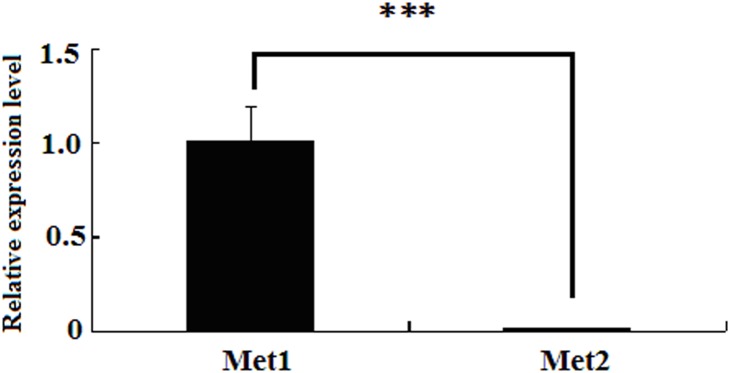
The relative expression level of two *Met* genes in PGs. PGs of newly emerged females were dissected, and total RNA was extracted for qPCR analysis. Rp49 gene was used as the housekeeping gene for normalization. The data represent the mean values ± SE of three biological replicates. Significance of comparisons are marked with *** (*p*<0.01) as determined by the Student’s *t*-test.

JH acts via Met, a putative JH receptor. Two *Met* genes, *Met1* and *Met2*, are present in *B. mori* [22]. The expression analysis using quantitative Real-time PCR (qPCR) manifested that the expression level of *Met1* was higher than that of *Met2* in the PGs (Fig. 4). This observation suggests that *Met1* plays an import role in JH signal in *B.mori* PGs.

### Injection of dsRNA and *in* *vivo* bombykol analysis

**Figure 5:**
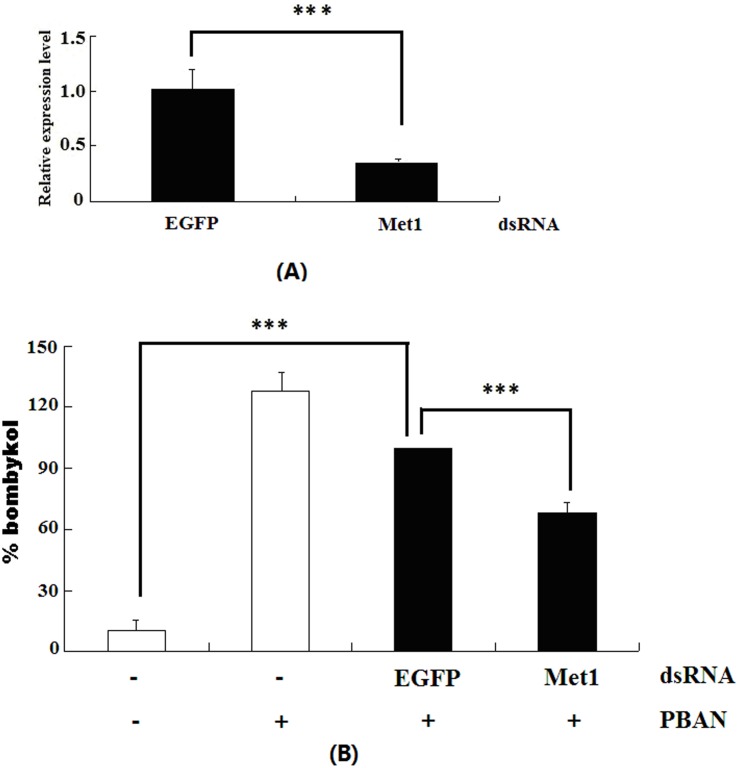
Effects of RNAi treatment on bombykol production. A: RNAi-induced reduction of Met1 gene. qPCR was carried out using cDNA generated from the total RNA extracted from PGs of females injected with 20 µg dsRNAs for *Met1* and *EGFP* (Control). B: Effects of RNAi for *Met1* on bombykol production. The females 48 h prior to eclosion were injected with double stranded RNAs, after 48 h waiting time, the females injected with dsRNA were decapitated and then were further injected with 10 pmol PBAN 24 h after decapitation. Bombykol production was measured by GC/MS from PGs 90 min after injection of PBAN. Bars indicate the mean values ± S.D. for independent experimental animals (n> = 12). Statistically significant differences from the PBAN alone are denoted by *** (*p*<0.01) as determined by the Student’s *t*-test.

RNAi-mediated knockdown of *Met1* was performed to confirm that the roles of JH in PBAN stimulated sex pheromone synthesis. Met1 dsRNA (15 µg) was injected into female pupae 48 h prior to eclosion and total RNA was extracted from PGs of 0 h females for qPCR analysis of target gene expression level. Results showed a significant decrease of *Met1* transcript compared with control injected with EGFP dsRNA (Fig. 5A).

After successful reduction of *Met1* mRNAs by RNAi, PBAN-induced bombykol production was determined by GC/MS. Results showed a significant reduction in bombykol production after *Met1* RNAi (Fig. 5B).

## Discussion

The suppression of female receptivity and sex pheromone production following mating is precisely regulated in most moths. Accessory-derived products and other seminal fluid proteins significantly change gene expression in females following mating, alterations in gene expression suppress female receptivity and sex pheromone production, ultimately leading to physiological and behavioral changes [23]–[26]. Many studies have focused on identifying seminal fluid components. SP is a seminal fluid component generated from the male accessory gland of *D. melanogaster*. this component is responsible for numerous post-mating responses, including promoted JH synthesis, enhanced egg laying and decreased female receptivity and sex pheromone [13]; [27]–[30]. The transfer of male factors triggers the neural inactivation of PBAN release and thus hinders the production of sex pheromone [7], [8]. During mating PGs are targeted by many male-originated factors such as SP and JH. As a result, the associated genes in PGs are inevitably activated. However, little is known about the effect of mating on gene expression in PGs. In the present study, we employed DGE profiling to elucidate the mating-regulated gene expression in PGs and the underlying molecular mechanisms. Eight PG libraries from mated and virgin females were constructed for the deep-sequencing analysis of gene expression patterns. The DGE search encompassed almost every transcript in the *B. mori* PG and revealed a corresponding set of differentially expressed gene in mated and virgin females. Thus, the putative genes involved in PG response to mating were defined.

The *B. mori* sex pheromone bombykol is derived from acetyl-CoA via fatty acid biosynthesis and subsequent modification of carbonyl carbon [5]. Prior to adult eclosion, PG cells rapidly generate and store abundant lipid droplets in the form of TAGs, the precursor of bombykol. Recent studies have elucidated in detail the mechanism underlying sex pheromone synthesis in *B. mori*. These studies have identified and characterized many genes. For example, fatty acid transport protein (BGIBMGA006185-TA) facilitates the uptake of extracellular long-chain fatty acids across the plasma membrane [31]. Fatty acid synthase (BGIBMGA004655-TA) and diacylglycerol o-acyltransferase (BGIBMGA008049-TA) are the key enzymes for fatty acid and TAG synthesis [20]. Perilipin (BGIBMGA013593-TA), triacylglycerol lipase (BGIBMGA005695-TA) and lipase (BGIBMGA008960-TA) play important roles in TAG lipolysis for bombykol release [1], [19], [32]. In the present DGE study, fatty acid transport protein, fatty acid synthase, diacylglycerol o-acyltransferase, triacylglycerol lipase and perilipin were all differentially up-regulated following mating, compared with the virgin female controls, suggesting that the PGs still maintain their capacity for sex pheromone synthesis and release after mating (Table 2). A previous study on *B.mori* showed that mated females still synthesize and release sex pheromone after PBAN stimulation [8]. Similarly, the PGs of mated *Choristoneura fumiferana* and *C. rosaceana* females are still capable of producing sex pheromone when stimulated by PBAN [33].

Mating also exhibits female adult immune reaction to male ejaculate [34]. In the present study, a lot of immune-associated genes [e.g., antimicrobial peptides (AMPs), peptidoglycan recognition protein and beta-1,3-glucan recognition protein 2] in PG cells were rapidly up-regulated 1 h after copulation (Table 2). Some of these genes continued to increase until 3 h- mating stage. PG is a bulbous valgus gland with an extrudable membrane, which was thought to be more vulnerable to pathogens or physically damage during the mating. Rapid activation of immune-associated genes during mating assists PGs in protecting against sexually transmitted pathogens or physical damages. Seminal fluid components transferred by male ejaculates are responsible for increased AMP gene expressions [34]. In *Drosophila*, SP is the major agent eliciting the transcription of AMPs by affecting the Toll and Imd pathways [35]. *B. mori* probably contains a SP-like seminal fluid component which induces AMP expression.

Most interestingly, DGE analysis indicated that the expression levels of some immune-associated genes (e.g., cecropin B, prophenoloxidase activating enzyme and lectin5) significantly decreased in females 24 h to 48 h after mating (Table 2). In *Tenebrio molitor*, mating elicits the immune suppression by decreasing of phenoloxidase activity [36]. Similarly, mating also reduces the number of hemocytes with encapsulation ability in the cricket *Altonemobius socius* [37]. The transfer of male sperm and seminal fluid proteins (e.g., SP) in *Drosophila* weakens female defense [38]. The phenomenon wherein the transcript levels of immune-associated genes increase in short-time mating and decrease in long-time mating can be attributed to the capacity of SP to rapidly increase the transcript in AMPs once mating. However, SP can also enhance JH production in the corpora allata, which usually functions as an immunosuppressor [39]. Despite their capacity to rapidly increase the levels of AMP transcripts in short-term mating (1 h and 3 h), SP or other seminal fluid proteins also stimulate the production of JH or other immune-suppressors production in long-term mating (24 and 48 h).

JH is a group of hormones that ensures larval growth while preventing metamorphosis. JH and 20-hydroxyrcdysone coordinately regulate molting and metamorphosis in insects. DGE analysis results indicated that JH signaling was activated leading to the up-regulation of a series of genes in the JH signaling pathway 24 h after mating (Table 2). The *Krüppel homolog 1* gene, a transcription factor induced by JH via a JH putative receptor (Met) was significantly up-regulated at later time after mating. This finding explains why immune suppression occurs at the later stage of mating. We further confirmed the effect of JH on sex pheromone biosynthesis in the newly emerged females. Results revealed that JH treatment significantly suppressed pheromone production in newly emerged females. The same suppression effect was elicited when newly emerged females were firstly decapitated to exclude the endogenous PBAN and then treated with JH and PBAN. These results suggested that JH inhibits the PBAN-induced sex pheromone biosynthesis in adult females (mature). We then examined the effect of inactivating the JH signal pathway on sex pheromone production in pupae. The two *Met* genes in the *Bombyx* genome are *Met1* and *Met2* [22]. The expression profile manifested that the transcript level of *Met1* was significantly higher than that of *Met2* in female PGs (more than 100 fold) (Fig. 4), suggesting that JH acts via *Met1* (not *Met2*) in female PGs. Further RNAi-mediated knockdown of *Met1* demonstrated that a decrease in the *Met1* transcript in pupae can significantly decrease in bombykol synthesis. This finding further proves that JH is necessary for sex pheromone synthesis in pupae and acts as a suppressor in adult females. Similarly, JH analogue initiates PBAN stimulation in pharate *H. armiger* females (1 day before emergence) but inhibits the level of expression of PBAN receptor gene in 1 day-old adult *H. armigera* females [13], [14], [40]. In *Drosophila*, corpora allata ablation delays the production of the major female sex pheromones, and lack of *Met* in Met (27) females delays the onset of mating [41]. Thus, JH modulates ovarian maturation and promotes sex maturation [42]. Moreover, JH titre increases 2 and 3d before eclosion in *B. mori* females for ovarian maturation [43]. In *Drosophila*, JH facilitates the maturation of female receptivity [44]. Hence, JH promotes sex maturation in females by acting as “age” hormone but suppresses sex pheromone production after sex maturation.

Some genes have been screened in PG cells after mating in the present study. However, the mechanism by which these mating-induced factors act and crosstalk in PG remains unclear. Future studies should focus on addressing these issues.

## Conclusions

Mating decreases female receptivity and terminates sex pheromone production in moths. PGs are targeted by many male-transferred factors. In this study, eight DGE libraries were constructed from the PGs of mating and virgin females. These libraries were used to investigate the gene expression profiles during mating. DGE profiling generated more than 3.4 million clean tags and revealed a series of genes showing differential expression in each pair of samples. Most importantly, JH signal was activated during mating. RNAi-mediated knockdown of JH receptor receptor gene *Met1* confirmed the functions of JH in sex pheromone synthesis. This study improves our understanding of the mechanism of the mating-elicited termination of sex pheromone production.

## Materials and Methods

### Insects

Larvae of *B. mori* (Zhenzhu×Chunlei) were maintained on mulberry leaves at 26°C under a 16∶8 light-dark cycle. Pupae were separated by gender, and males and females were stored in separate cages until emergence.

Newly emerged virgin females were easily mated when placed together with the males. The mating usually continued to proceed until artificial termination.

### Chemicals

PBAN of *B. mori* was synthesized by Sangon Biotech (Shanghai) Co., Ltd. The sex pheromone component, Bombykol, was obtained from Shogo Matsumoto (RIKEN, Advanced Science Institute, Japan) and used as the internal standard for GC/MS.

### Sample collection and RNA extraction

Newly emerged virgin females were selected to mate with males. The effects of mating on bombykol production contain early- and later-stage. A mating duration of more than 6 h was thought to permanently terminate the bombykol production in *B.mori* according to previous study [8]. Thus early mating time (1 h and 3 h) and later mating time (24 h and 48 h) were selected to investigate the effect of mating on gene expression in the pheromone glands. PGs were dissected at different mating times of 1, 3, 24 and 48 h and were designated as M1, M3, M24 and M48 receptively. The corresponding PGs from the virgin females were used as controls and designed as V1, V3, V24 and V48. The samples were immediately stored at –80°C for later use.

Total RNA was extracted from the collected samples using Trizol reagent (Invitrogen) according to the manufacturer’s instructions. RNA concentration was further confirmed by measuring the absorbance at 260 nm on a spectrophotometer.

### DGE library preparation and sequencing

Eight DGE libraries (M1, M3, M24, M48, V1, V3, V24 and V48) were prepared by using the Illumina gene expression sample prep kit. Briefly, mRNA from eight samples was captured with Oligo(dT) magnetic beads from total RNA and the first and second-strand cDNA fragments were synthesized. The bead-bound cDNA was subsequently digested with the restriction enzyme *Nla*III. cDNA fragments with 3′ ends were purified through bead precipitation, and the Illumina adaptor 1 was added to the sticky 5′ end of these cDNA fragments. The junction of the Illumina adaptor 1 and the CATG site was the recognition site of *Mme*I, which cuts at 17 bp downstream of the CATG site. Moreover 21 base-pair tags containing adaptor 1 were produced. After removing the 3′ fragments by magnetic bead precipitation, Illumina adaptor 2 was introduced to the 3′ ends of the tags to obtain a tag library. After 15 cycles of polymerase chain reaction (PCR) amplification, 85 bp fragments were purified by 6% Tris/Borate/EDTA-polyacrylamide gel electrophoresis. The single-chain nucleotides were fixed onto an Illumina sequencing chip (flow cell) after denaturation. Adaptor 1 was used as the sequencing primer. Each tunnel generated millions of raw reads with a sequencing length of 35 bp.

### Aligning DGE tags to reference genomic data

Sequencing-received raw image data were transformed by base calling into sequence data, known as raw data or raw reads. Prior to mapping the DGE tags, low-quality tags, including adaptor sequences, tags with unknown nucleotide “N”, empty tags (only adaptor sequence), and tags with one copy number (probably from sequencing errors) were filtered after data processing. All possible CAGT+17 nucleotide tags were established by using the *Bombyx* genomic database and other NCBI data. All clean tags were mapped to the reference sequences, and only one nucleotide mismatch was allowed. Clean tags mapped to the reference sequences from multiple genes were filtered. Index of the reference genome was built using Bowtie v 0.12.8 and paired-end clean reads were aligned to the reference genome using TOPHat v1.4.0. The remaining clean tags were designated as unambiguous clean tags. The number of unambiguous clean tags for each gene was calculated and then normalized to number of transcripts per million clean tags (TPM) [16], [17].

### Evaluation of DGE libraries

The differences in gene expression in different PG libraries (M1/V1, M3/V3, M24/V24 and M48/V48) were compared by statistically analyzing the tag frequency in each DGE library according to the method described by Audic *et al*.[18]. The false discovery rate (FDR) was used to determine the threshold P-value in multiple tests and analysis. FDR<0.001 and an absolute value of the log2 ratio >1 were used as the thresholds to determine significant differences in gene expression.

Differentially expressed genes were selected for further Gene Ontology (GO) and KEGG Orthology (KO) enrichment analyses based on hypergeometric tests. The calculating formula was
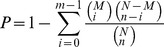
Where *N* represents the number of genes with GO/KO annotation, *n* represents the number of DGEs in *N*, *M* represents the number of genes in each GO/KO term, and *m* represents the number of expressed gene in each GO/KO term. A corrected P-value<0.05 was selected as a threshold for significant enrichment of the gene sets. In contrast, for KO enrichment analysis, FDR<0.05 was used as the threshold to determine significant enrichment of the gene sets.

### Effect of JH on bombykol production

Newly emerged virgin females were treated with JH analogues (methoprene) for 2 h, and then injected with 10 pmol PBAN for 90 min. The PGs were removed and extracted in hexane for further GC/MS analysis after the treatment. Acetone was used as the negative control.

Newly emerged virgin females were decapitated. After 24 h of waiting time, the decapitated females were treated with methoprene for 2 h and then injected with 10 pmol PBAN for 90 min. The PGs were dissected and extracted in hexane for further GC/MS analysis. Acetone was used as the negative control.

### Double-stranded RNA (dsRNA) synthesis

The synthesis of dsRNAs was performed using the MEGAscript RNAi kit (Ambion) according to the manufacturer’s instructions. The templates for dsRNA synthesis were prepared by PCR using gene-specific primers containing T7 polymerase sites as previously described [19], [20]. All the primer sets are listed in Table S6. PCR was performed under the following conditions: 94°C for 3 min, followed by 35 cycles of 94°C for 1 min, 59°C for 1 min, and 72°C for 1 min, and a final elongation at 72°C for 10 min. The PCR products were purified using a PCR product purification kit. The purified PCR product was used as template for *in* *vitro* dsRNA synthesis. After removing the template DNA and single-stranded RNA by DNase and RNase treatments respectively, dsRNA was purified using MEGAclearTM columns (Ambion) and then eluted in diethyl pyrocarbonate-treated nuclease-free water. The dsRNA concentrations were measured using a biophotometer (Eppendorf). The dsRNA of enhanced green fluorescent protein (EGFP) was used as the negative controls.

The effects of RNAi on the transcript expression were analyzed by using qPCR. The primers used in this experiment are shown in Table S5.

### Injection of dsRNA and *in* *vivo* bombykol analysis

Fifteen micrograms of dsRNA were injected to the abdominal intersegment membrane of female pupae at 48 h prior to eclosion. The females were maintained until emergence. The newly emerged females were decapitated, maintained at normal condition for 24 h and then injected with 10 pmol PBAN. The PGs were dissected 90 min after the injection and then dissolved in hexane. Control females were injected with dsRNA of EGFP.

Bombykol accumulation was measured by GC/MS (Trace GC Ultra Trace DSQ; MS-Thermo Scientific DSQ II) equipped with a 30 m capillary column (RTX-5SILMS, Restek, 0.25 mm diameter). Each sample containing a pooled hexane extract from 12 or more *B. mori* PGs was subjected to GC/MS analysis.

### Quantitative Real-time PCR (qPCR)

The first-strand cDNA was synthesised from the total RNA (1 µg) from each sample using the PrimeScript RT reagent kit with gDNA Eraser (TaKaRa). The primers designed for qPCR analysis are listed in Table S5. *Bombyx* ribosomal protein 49 (rp49) gene was used as an internal control gene for normalization. qPCR was performed using SYBR green supermix (TaKaRa) according to the manufacturer’s instructions. The thermal cycle conditions for the qPCR were 95°C for 10 min, followed by 40 cycles of 95°C for 15 s and 60°C for 1 min. The specificity of the SYBR green PCR signal was further confirmed by melting curve analysis and agarose gel electrophoresis. The mRNA expression was quantified using the comparative CT (Cross Threshold, the PCR cycle number that crosses the signal threshold) method [21]. The CT of the rp49 gene was subtracted from the CT of the target gene to obtain ΔCT. The normalized fold changes of the target gene mRNA expression were expressed as 2^−ΔΔCT^, where ΔΔCT is equal to ΔCT_ treated sample_ –ΔCT_ control_.

### Statistical analysis

qPCR and GC/MS experiments were performed in triplicate. Results are expressed as means±standard deviation (SD). qPCR and GC/MS results were compared using student's *t-*tests.

## Supporting Information

Figure S1 **Different components of the raw tags in each sample.** The percentages of clean tags, raw tags containing N, empty tags with adaptor only, and tags with copy number <2.
(TIF)

Figure S2 **Distribution of total clean tags and distinct clean tags in each sample. A**: Distribution of total clean tags. **B**: Distribution of distinct clean tags.
(TIF)

Figure S3 **Gene expression level in each comparison.** “Not DEGs” indicates “not detected expression genes”. X-axis and Y-axis present log10 of the transcript per million of differentially developmental stages of PGs. *p*< = 0.001 and absolute value of log2> = 1 were used as the thresholds.
(TIF)

Figure S4 **GO categories of each comparison.**
(TIF)

Table S1 **Differentially expressed genes between M1 and V1.**
(XLS)

Table S2 **Differentially expressed genes between M3 and V3.**
(XLS)

Table S3 **Differentially expressed genes between M24 and V24.**
(XLS)

Table S4 **Differentially expressed genes between M48 and V48.**
(XLS)

Table S5 **Primers used in real-time PCR and RNAi effect analysis.**
(DOC)

Table S6 **Primers used in dsRNA synthesis.**
(DOC)
