# Supplementary figures and images for: Identification of Differentially Expressed Genes in the Pheromone Glands of Mated and Virgin Bombyx mori by Digital Gene Expression Profiling
Source: PLoS One. 2014 Oct 20;9(10):e111003. doi: 10.1371/journal.pone.0111003 (PMC4203833; doi:10.1371/journal.pone.0111003)

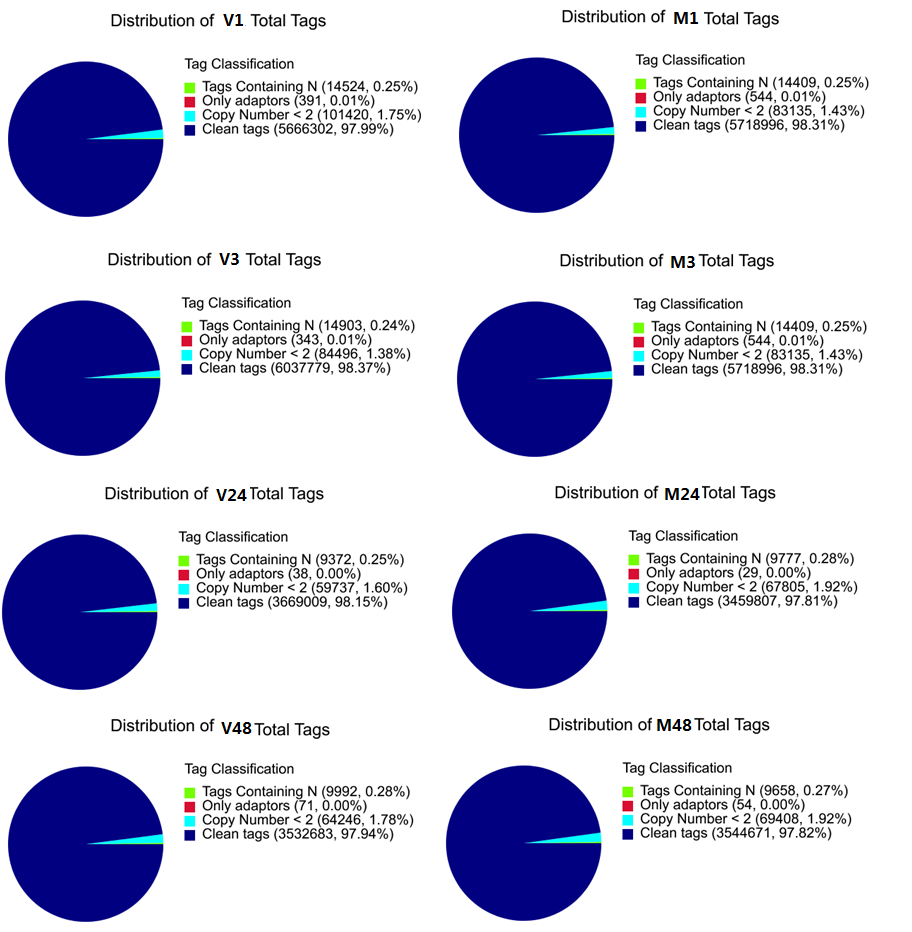

Supplement: Figure S1 — Different components of the raw tags in each sample. The percentages of clean tags, raw tags containing N, empty tags with adaptor only, and tags with copy number <2. (TIF) [file pone.0111003.s001.tif]

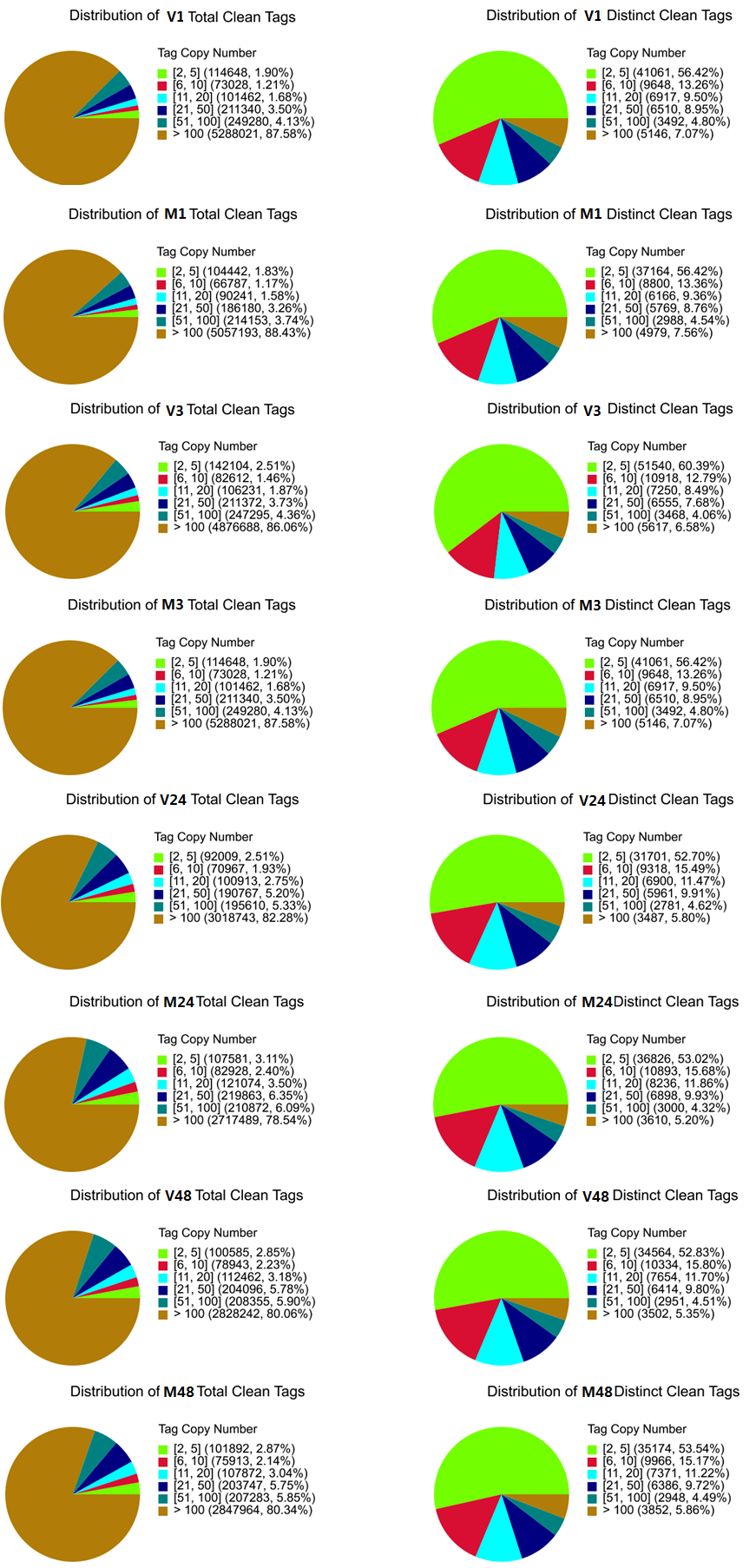

Supplement: Figure S2 — Distribution of total clean tags and distinct clean tags in each sample. A: Distribution of total clean tags. B: Distribution of distinct clean tags. (TIF) [file pone.0111003.s002.tif]

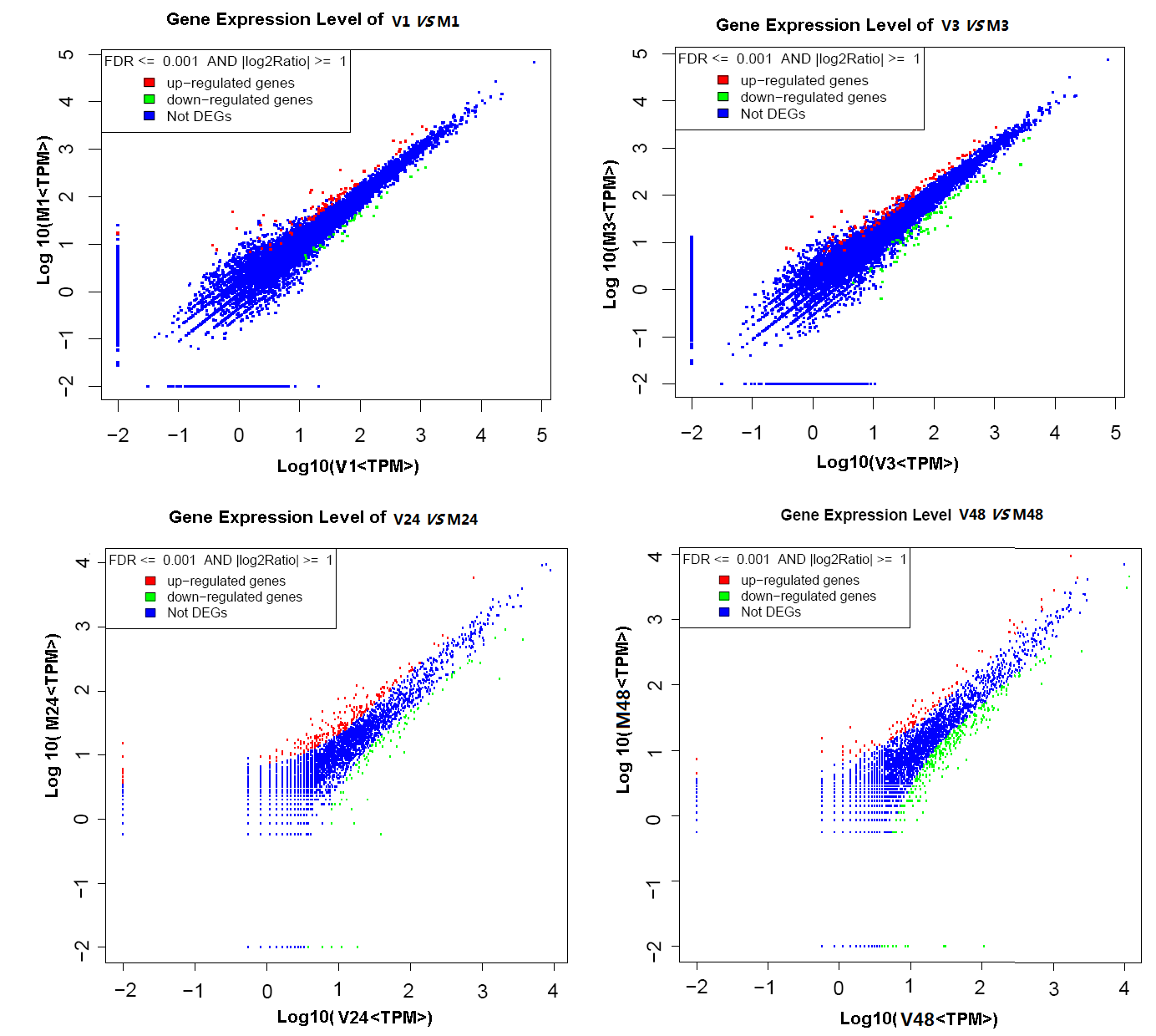

Supplement: Figure S3 — Gene expression level in each comparison. “Not DEGs” indicates “not detected expression genes”. X-axis and Y-axis present log10 of the transcript per million of differentially developmental stages of PGs. p< = 0.001 and absolute value of log2> = 1 were used as the thresholds. (TIF) [file pone.0111003.s003.tif]

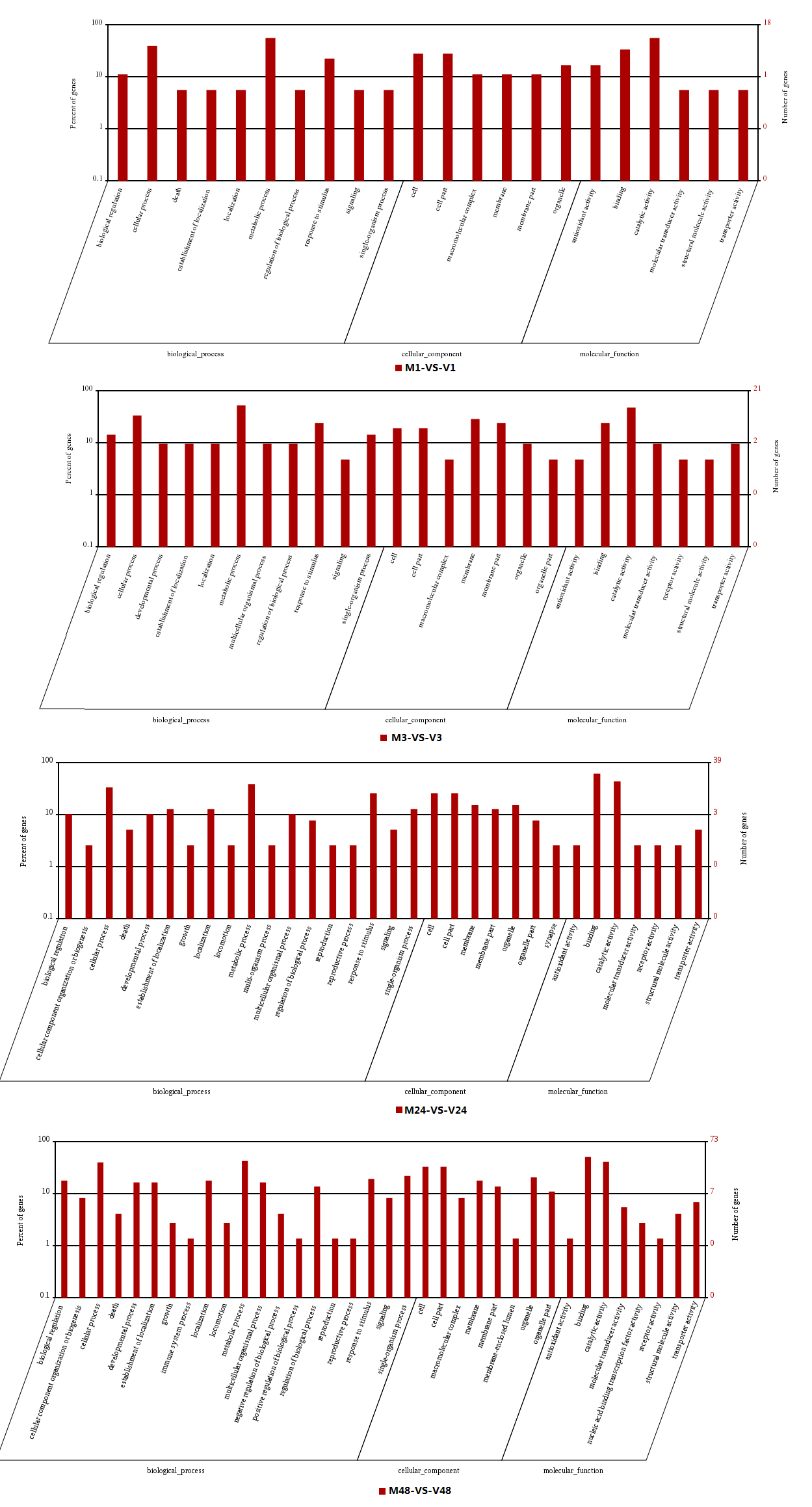

Supplement: Figure S4 — GO categories of each comparison. (TIF) [file pone.0111003.s004.tif]
