# Supplementary material for: Identification of Differentially Expressed Genes in the Pheromone Glands of Mated and Virgin Bombyx mori by Digital Gene Expression Profiling
Source: PLoS One. 2014 Oct 20;9(10):e111003. doi: 10.1371/journal.pone.0111003 (PMC4203833; doi:10.1371/journal.pone.0111003)
Supplement: Table S5 — Primers used in real-time PCR and RNAi effect analysis. (DOC) [file pone.0111003.s009.doc]

**Table S5 List of primers for Real-time PCR and RNAi effect analysis**

| **Gene** | **Forward primer (5′–3′)** | **Reverse primer (5′–3′)** |
| --- | --- | --- |
| Met1 | ATGTGCGTTGGGTTATCTGT | TTCATTTCTTCCATCATTCT |
| Met2 | CACATCGAAGGCTCATTCAG | GATCTGTCCATCGACAGAGTG |
| Rp49 | CAGGCGGTTCAAGGGTCAATAC | TGCTGGGCTCTTTCCACGA |

Rp49: ribosomal protein 49
