# Supplementary material for: Identification of Differentially Expressed Genes in the Pheromone Glands of Mated and Virgin Bombyx mori by Digital Gene Expression Profiling
Source: PLoS One. 2014 Oct 20;9(10):e111003. doi: 10.1371/journal.pone.0111003 (PMC4203833; doi:10.1371/journal.pone.0111003)
Supplement: Table S6 — Primers used in dsRNA synthesis. (DOC) [file pone.0111003.s010.doc]

**Table S6 List of primers for dsRNA synthesis**

| **Gene** | **Forward primer（5′→3′）** | **Reverse primer（5′→3′）** |
| --- | --- | --- |
| Met1 | GATCACTAATACGACTCACTATAGGGAGACCGTCAACAAACTGCGTAAC | GATCACTAATACGACTCACTATAGGGAGAGGATGATCAGACCTTGCTCC |
| EGFP | GATCACTAATACGACTCACTATAGGGAGACCTGAAGTTCATCTGCACCAC | GATCACTAATACGACTCACTATAGGGAGACTCCAGCAGGACCATGTGATC |
